# Supplementary material for: A Comprehensive Public Health Evaluation of Lockdown as a Non-pharmaceutical Intervention on COVID-19 Spread in India: National Trends Masking State Level Variations
Source: medRxiv. 2020 Jun 14:2020.05.25.20113043. Preprint. [Version 2] doi: 10.1101/2020.05.25.20113043 (PMC7310653; doi:10.1101/2020.05.25.20113043)
Supplement: Supplement 2020 [file 85905-2020.05.25.20113043-1.docx]

**Supplementary information for**

**A Comprehensive Public Health Evaluation of Lockdown as a Non-pharmaceutical Intervention on COVID-19 Spread in India: National Trends Masking State Level Variations**

Deepankar Basu^1^, PhD; Maxwell Salvatore^2,3^, MPH; Debashree Ray^4,5^, PhD; Mike Kleinsasser^2^, MS; Soumik Purkayastha^2^, MS; Rupam Bhattacharyya^2^, MS; Bhramar Mukherjee^2,3^*, PhD.

^1^: Department of Economics, University of Massachusetts, Amherst, MA 01002, USA

^2^: Department of Biostatistics, University of Michigan, Ann Arbor, MI 48109, USA

^3^: Center for Precision Health Data Science, University of Michigan, Ann Arbor, MI 48109, USA

^4^: Department of Epidemiology, Johns Hopkins University, Baltimore, MD 21205, USA

^5^: Department of Biostatistics, Johns Hopkins University, Baltimore, MD 21205, USA

*: Corresponding author. Address - Department of Biostatistics, School of Public Health, University of Michigan, 1420 Washington Heights, Ann Arbor, MI 48109-2029, USA. Telephone – (734) 764-6544. Email – [bhramar@umich.edu](mailto:bhramar@umich.edu).

Table of Contents

[SUPPLEMENTARY METHODS 3](#_Toc42798202)

[**Estimation and Confidence Interval (CI) for Case-Fatality Rates** 3](#_Toc42798203)

[**Doubling Time** 3](#_Toc42798204)

[**Time-Varying R Estimates** 4](#_Toc42798205)

[**Test Positive Rate** 5](#_Toc42798206)

[**Testing Shortfall** 5](#_Toc42798207)

[REFERENCES 6](#_Toc42798208)

[SUPPLEMENTARY FIGURES 7](#_Toc42798209)

[**Supplementary Figure 1**. Cumulative number of reported cases, fatalities, and recovered cases in India over the period between March 15 and May 31. 7](#_Toc42798210)

[**Supplementary Figure 2**. Cumulative number of reported COVID-19 cases in 20 Indian states and union territories over the period between March 15 and May 31. 8](#_Toc42798211)

[**Supplementary Figure 3**. Cumulative number of reported COVID-19 deaths in 20 Indian states and union territories over the period between March 15 and May 31. 8](#_Toc42798212)

[**Supplementary Figure 5**. Forest plot of estimated case-fatality rates based on closed cases only as of May 31, along with 95% confidence intervals, for 20 states and union territories of India, and a national summary. 10](#_Toc42798213)

[SUPPLEMENTARY TABLES 11](#_Toc42798214)

[**Supplementary Table 1**. Description of total tests, population tested, and test-positive rate by state/union territory and nationwide as of May 31. The states that need improvement (defined as having test-positive rate >6%) are in red. 11](#_Toc42798215)

# SUPPLEMENTARY METHODS

## **Estimation and Confidence Interval (CI) for Case-Fatality Rates**

Let us denote the cumulative number of confirmed cases and deaths for a region of interest (India or one of the states/union territories) at a given date (May 31 for our purpose) respectively by $C$ and $D$. Assuming that the proportion of underreporting (due to impossibility of testing all cases and imperfection of the tests) in the fatal and non-fatal cases are same, $D|C\sim Bin(C,\pi)$ where $\pi$ is the true underlying case-fatality ratio. Therefore, assuming sufficiently large number of cases, via central limit theorem, we can write $\sqrt{C}\left( \hat{\pi}-\pi\right)\sim AN\left( 0,\pi\left( 1-\pi\right) \right)$, where  $\hat{\pi}=\frac{D}{C}$. Using delta method on this, we get $\sqrt{C}\left( logit\left( \hat{\pi} \right)-logit\left( \pi\right) \right)\sim AN\left( 0,\frac{1}{\pi\left( 1-\pi\right)} \right)$, where $logit\left( x \right)=\log\left( \frac{x}{1-x} \right)$. Therefore, one estimator the standard deviation of $\hat{\pi}$ is given by $s=\sqrt{\frac{1}{C\hat{\pi}(1-\hat{\pi})}}=\sqrt{\frac{C}{D(C-D)}}$. Using this, we can get a 95% CI for $logit(\pi)$ as $(logit\left( \hat{\pi} \right)\pm z_{0.975}s)$. Inverting this by applying the function $expit\left( x \right)=\frac{e^{x}}{1+e^{x}}$, we get a 95% CI for $\pi$.

It is important to note that this method inherently assumes that all events (deaths/recoveries) that could possibly happen from the set of observed confirmed cases has happened by the day on which the data is observed, which of course is not true in general. One standard alternative approach here is to look at the closed cases only. Assume that the cumulative number of recovered cases at the same date for the same region as before is denoted by $R$. Then, using $D+R$ in place of $C$ in the above calculations throughout, we can get another estimate and CI for the true case-fatality rate (CFR2, ratio of the total number of deaths and the sum of the same and the total number of recovered cases).

## **Doubling Time**

We calculate doubling time, $T_{d}$, assuming a constant growth rate $r$% within time $t$ using the formula

$$T_{d}=t\frac{\ln\left( 2 \right)}{ln(1+ r)},$$

where $r$ is calculated as

$$r= \frac{T_{end}-T_{start}}{T_{start}}.$$

We calculated the doubling time using a trailing 7-day window, i.e., the doubling time for May 7 represents how long cases would take to double assuming a constant growth in cases from May 1 to May 7.

## **Time-Varying R Estimates**

We estimate the effective reproduction number for COVID-19 in India using the EpiEstim package in R and data from COVID-19 India, a crowdsourced effort that relies on volunteer validation of state bulletins and official handle reports.^1, 2^ We refer to the effective reproduction number as “R” throughout, which is similar to the concept of R_0_, however, R_0_ is a constant that is inherent to the pathogen and is not time-variant or impacted by interventions (such as social distancing or lockdown). This instantaneous R is recommended for evaluating effective control measures.

We use the “parametric_SI” estimation method and a 5-day window (“estimate_R” function, which was used to describe the progression of the outbreak in Wuhan).^1, 3^ We also use a gamma distribution prior with a mean of 7 days and a standard deviation of 4**.**5 days, based on research by Wu and colleagues, for the generation time (a distribution of the onset of disease used to estimate R).^4^

We looked at the effective reproduction number for COVID-19 nationwide in India using data from March 1 to May 18. Because the estimation requires several days of data for reliable, consistent results, we only observe data from March 15 to May 18. We also estimated R over the time period for the 20 states/union territories with the greatest number of total reported cases as of May 18. State-level data was first reported by COVID-19 India on March 15 and we begin the plots on March 24 to allow the estimates to stabilize.^2^ There are some states/union territories for which the first cases were not reported until after March 24 (e.g., Tripura), in which case we see the initial elevated R estimates because the estimates have not yet stabilized.

We see that the estimated R varies across states/union territories and, in some cases, does drop below one (indicated by the dashed horizontal lines in Figures 3 and 5). It is worth noting that in several of these cases, it returns to above 1 after it drops below 1, highlighting that, despite time-varying estimates, no state/union territory is in the clear yet. The plots report the average R and 95% CIs *for the past 7 days* corresponding to the highlighted state/union territory.

## **Test Positive Rate**

The test positive rate was calculated as the ratio of cumulative reported number of positive tests to the reported total number of tests on a given date (COVID-19 India state-level testing data begins April 1).^2^ While COVID-19 India also has national-level testing data, it is spotty, and in recent weeks, have not been reporting the number of positive tests. As such, for national test-positive rates, we sum the positive tests and total tests over all the 35 states and union territories for which data were reported for national counts and rates. It will be to acquire consistency across data sources on the testing data.

## **Testing Shortfall**

The testing shortfall is a metric used to estimate the increase in the number of tests that should be seen relative to a 2% benchmark test positive rate. First, we calculate the desired number of tests, $T_{D}$:

$$T_{D}=\frac{TPR_{O}}{TPR_{D}}T_{O}$$

Where $TPR_{0}$ is the 7-day average of the observed, cumulative test-positive rate, $TPR_{D}$ is the target test-positive rate (in this case $TPR_{D}=0.02$, and $T_{O}$ is the observed number of cumulative tests.

With this value, we calculate the shortfall, or the number of additional total tests required to achieve the test-positive rate as:

$$shortfall=\max\left( T_{D}-T_{O}, 0 \right).$$

When shortfall is equal to 0, the number of tests being performed is theoretically sufficient given the number of cases being observed. When shortfall is greater than 0, it represents the number of additional tests that should be performed given the number of cases being observed.

# REFERENCES

1. Cori A, Ferguson NM, Fraser C, Cauchemez S. A new framework and software to estimate time-varying reproduction numbers during epidemics. American journal of epidemiology. 2013; 178(9):1505-12.

2. COVID-19 India. COVID-19 Tracker Updates for India for State-wise and District-wise data. 2020. Retrieved May 2020, from COVID19 India: https://www.covid19india.org/.

3. Pan A, Liu L, Wang C, Guo H, Hao X, Wang Q, et al. Association of public health interventions with the epidemiology of the COVID-19 outbreak in Wuhan, China. Jama. 2020.

4. Wu JT, Leung K, Bushman M, Kishore N, Niehus R, de Salazar PM, et al. Estimating clinical severity of COVID-19 from the transmission dynamics in Wuhan, China. Nature Medicine. 2020; 26(4):506-10.

# SUPPLEMENTARY FIGURES

## **Supplementary Figure 1**. Cumulative number of reported cases, fatalities, and recovered cases in India over the period between March 15 and May 31.

## **Supplementary Figure 2**. Cumulative number of reported COVID-19 cases in 20 Indian states and union territories over the period between March 15 and May 31.

## **Supplementary Figure 3**. Cumulative number of reported COVID-19 deaths in 20 Indian states and union territories over the period between March 15 and May 31.

**Supplementary Figure 4**. Time series plots of test positive rates for 20 Indian states and union territories.

## **Supplementary Figure 5**. Forest plot of estimated case-fatality rates based on closed cases only as of May 31, along with 95% confidence intervals, for 20 states and union territories of India, and a national summary.

# SUPPLEMENTARY TABLES

## **Supplementary Table 1**. Description of total tests, population tested, and test-positive rate by state/union territory and nationwide as of May 31. The states that need improvement (defined as having test-positive rate >6%) are in red.

| **Location** | **Total tests** | **Population tested (%)** | **Test-positive rate (%)** |
| --- | --- | --- | --- |
| Andhra Pradesh | 372748 | 0.75 | 0.96 |
| Assam | 109097 | 0.35 | 1.23 |
| Bihar | 75737 | 0.07 | 5.03 |
| **Delhi** | **212784** | **1.27** | **9.33** |
| **Gujarat** | **211930** | **0.35** | **7.92** |
| Haryana | 118138 | 0.47 | 1.77 |
| Jammu and Kashmir | 171045 | 1.37 | 1.43 |
| Jharkhand | 65886 | 0.20 | 0.96 |
| Karnataka | 293575 | 0.48 | 1.10 |
| Kerala | 77508 | 0.23 | 1.64 |
| Madhya Pradesh | 167808 | 0.23 | 4.82 |
| **Maharashtra** | **463177** | **0.41** | **14.61** |
| Odisha | 152131 | 0.36 | 1.28 |
| Punjab | 87852 | 0.32 | 2.58 |
| Rajasthan | 409777 | 0.60 | 2.16 |
| Tamil Nadu | 491962 | 0.68 | 4.54 |
| **Telangana** | **23388** | **0.07** | **11.54** |
| Uttar Pradesh | 289892 | 0.15 | 2.79 |
| Uttarakhand | 30438 | 0.30 | 2.98 |
| West Bengal | 203751 | 0.22 | 2.70 |
| **India** | **3737027** | **0.28** | **5.10** |
